# Pause before action: Waiting short time as a simple and resource-rational boost

**DOI:** 10.1038/s41598-025-87119-z

**Published:** 2025-02-05

**Authors:** Masaru Shirasuna, Rina Kagawa, Hidehito Honda

**Affiliations:** 1 Faculty of Psychology, Otemon Gakuin University, 2-1-15, Nishiai, Ibaraki-Shi, Osaka, 567-8502 Japan; 2 Faculty of Informatics, Shizuoka University, 3-5-1, Johoku, Chuo-Ku, Hamamatsu-Shi, Shizuoka, 432-8011 Japan; 3 Institute of Medicine, University of Tsukuba, 1-1-1 Tennoudai, Tsukuba-Shi, Ibaraki, 305-8575 Japan

**Keywords:** Speed-accuracy trade-off, Resource rationality, Thinking costs, Boost, Judgment accuracy, Psychology, Human behaviour

## Abstract

Many workers today engage in straightforward judgment tasks, increasing the need for interventions to improve accuracy. We propose a resource-rational and psychohygienic intervention, “wait short time”, which introduces a brief pause before displaying alternatives. This pause works as a harmonious triad: it clears the mind of prior judgment bias, restores present attention, and prepares the mind for future judgments; and all this without additional instructions. Based on a resource rationality framework, cognitive costs (e.g., irritation, cognitive conflict) are associated with prolonged thinking because of humans limited cognitive resources. Therefore, there should be an appropriately short thinking time to achieve higher accuracy with minimal workload. We investigated the effectiveness of the proposed intervention both theoretically and empirically. The computer simulations demonstrated that, under assumptions of limited cognitive resources, there was an optimal time at the early stages for maximizing total benefits. The results of behavioral experiment was consistent with the theoretical findings: Providing a waiting time (1 s or 2.5 s) improved judgment accuracy, but cognitive conflicts increased over time and an unnecessarily long time (2.5 s) induced more subjective irritation. Consequently, an appropriate time (1 s) could enhance judgment accuracy with less workload. We discuss the implications and limitations of the proposed intervention.

## Introduction

Recently, the number of online workers such as crowdsourcing platforms has increased worldwide. They often conduct various types of simple binary choice tasks to create learning data for machine learning, including data annotation. In such contexts, there is a growing need for interventions to improve workers’ judgments accuracy. Recent cognitive science studies have proposed an intervention framework called *boost*^1,2^, which aims to change people’s judgments and behaviors toward a desirable direction by fostering cognitive competence. Regarding a behavioral change intervention, *nudge*^3,4^ is well-known and also aims to change people’s behaviors by manipulating choice architecture^3,5,6^. However, nudge is sometimes criticized in terms of disregarding workers’ autonomy and the temporal duration of the effects^7^. In contrast to the nudge approach, the boost approach focuses on fostering people’s cognitive competence such as their ability to perform tasks by a simple intervention (e.g., in Bayesian probability judgments, simply manipulating the information presentation format presented, such as changing information from “30%” to “30 out of 100 people”, enhanced judgment accuracy^8,9^). By fostering competence, boost is expected to retain worker autonomy and provide long-lasting effects after the intervention is removed^1^. In the context of public workers, it will be possible that judgment accuracy can be improved by a simple and low-cost intervention.

We propose a “wait short time” intervention to foster workers’ competence, involving inserting an appropriately short time (e.g., 1 s) before presenting alternatives to afford workers a time to think, without explicit instructions. This intervention may prove useful in various task domains, because it is relatively simple and low-cost and does not impose much cognitive load.

Regarding the relationship between thinking time and judgment accuracy, *speed-accuracy trade-off* (SAT) is a well-known framework in the contexts of information processing, judgment and decision-making^10–12^. The SAT assumes that faster responses mean less accumulated evidence and a highly likely to generate less accurate judgments, and vice versa. The extent of the increase in accuracy is remarkable early on but gradually diminishes as time elapses. The SAT process can be intuitively understood in terms of sequential sampling (or evidence accumulation) models^13–15^. Sequential sampling models assume that individuals set a decision threshold during a choice task and make a final judgment when the amount of evidence (started from a certain baseline) reaches the threshold. The models also posit that individuals sometimes adjust their decision thresholds and baseline. That is, lowering the threshold or raising the baseline leads to faster responses but simultaneously to increase error rates because of the lower amount of accumulated evidence, and vice versa (e.g.^16^). It is interpreted that longer thinking time leads to better stimulus encoding and then enables individuals to make better judgments.

In SAT, a longer thinking time is assumed to generate better accuracy. In the real world, however, human cognitive resources (e.g., computational capacity and information storage) are limited and cannot handle large amounts of data. Therefore, thinking itself is considered costly for humans^17,18^. While a longer thinking can yield greater judgment accuracy, it can also generate a larger cognitive load (e.g., subjective irritation because of extra thinking time, cognitive conflicts between different alternatives).

Recent cognitive science studies have proposed a *resource rationality* framework^19–21^. Resource-rational approaches are key to model human minds because the approaches consider our limited cognitive resources (see also *bounded rationality* in^22,23^). Resource-rational behaviors are modeled in terms of value of computation (VOC): Humans behave to maximize VOC, which is roughly described as the expected utility or reward of making decisions minus the expected cost of time. If we reflect this framework in the SAT context, we can consider that the increase of accuracy by thinking as expected reward (we refer “thinking benefit”), the increasing cognitive load by thinking as “thinking cost”, and the difference between them (i.e., something like “overall goodness” under the trade-off between accuracy and costs) as VOC (we refer “total benefit”).

SAT generally assumes that judgment accuracy can be maximized through taking sufficient time to think. In the context of SAT, the aspects of cognitive resources have been ignored because SAT has been discussed so far mainly based on judgment accuracy. That is, thinking benefit is often regarded as equivalent to total benefit. In contrast, resource rationality argues that total benefits obtained from tasks should be considered based on the trade-off between thinking benefits (i.e., accuracy increase) and thinking costs (i.e., cognitive load increase). As a person spends more time considering a task, the extent of increase in judgment accuracy gradually diminishes, and it is unlikely to further improve accuracy after a certain time point (i.e., judgment accuracy reaches a peak at a certain point, not to continue to increase accuracy over time); instead, thinking costs continue to linearly increase. This implies that the total benefits gradually decrease after the peak of accuracy. Thus, affording a long (an unnecessarily long) time will not always be a good intervention.

These considerations bring forwards the following hypothesis: There is an appropriately short time which enables the total benefit peak (i.e., higher thinking benefits with relatively lower thinking costs) to be achieved. In addition, if there is such an appropriate thinking time, it is predicted that an intervention that provides an appropriately short thinking time (“wait short time”) will be effective, rather than providing a long thinking time. We aim to theoretically and empirically demonstrate this prediction from the framework of resource rationality. Specifically, under assumptions of limited cognitive resources (i.e., cognitive costs will increase over time), we show that there should be an “optimal” thinking time through computer simulations and that such tendencies are observed even actual human behaviors through behavioral experiment. Here, we considered that our arguments described above are not necessarily in opposition to the SAT framework; in contrast, we believe that our study will have potential to provide a new insight for SAT.

Several studies indicate that a brief waiting time (or pause) can improve judgment performance. For example, global and local pauses each have distinct benefits, particularly for complex over simple, speeded decisions^24^. In risky choice tasks, short pauses led individuals to choose higher expected value options more frequently^25^. Pauses could also reduce misinformation sharing on social media^26,27^. Yet, these effects remain unexplored in terms of human cognitive factors, such as resource rationality.

The outline of this study is as follows. Using a binary choice task, we examined whether our proposed “wait short time” intervention can effectively boost performance under humans’ limited cognitive resources. In Study 1, through computer simulations assuming humans limited cognitive resources, we theoretically demonstrate that there should be a peak in total benefits for a judgment task under the trade-off between thinking benefits and thinking costs. In Study 2, through behavioral experiment confirming whether the theoretical findings are consistent with actual human behaviors, we empirically investigated the effectiveness of the intervention by manipulating waiting times.

## Study 1: Computer simulations

First, we conducted computer simulations to theoretically examine the differences in benefits and costs between SAT and resource rationality.

### Methods

#### Tasks and materials

**Fig. 1:**
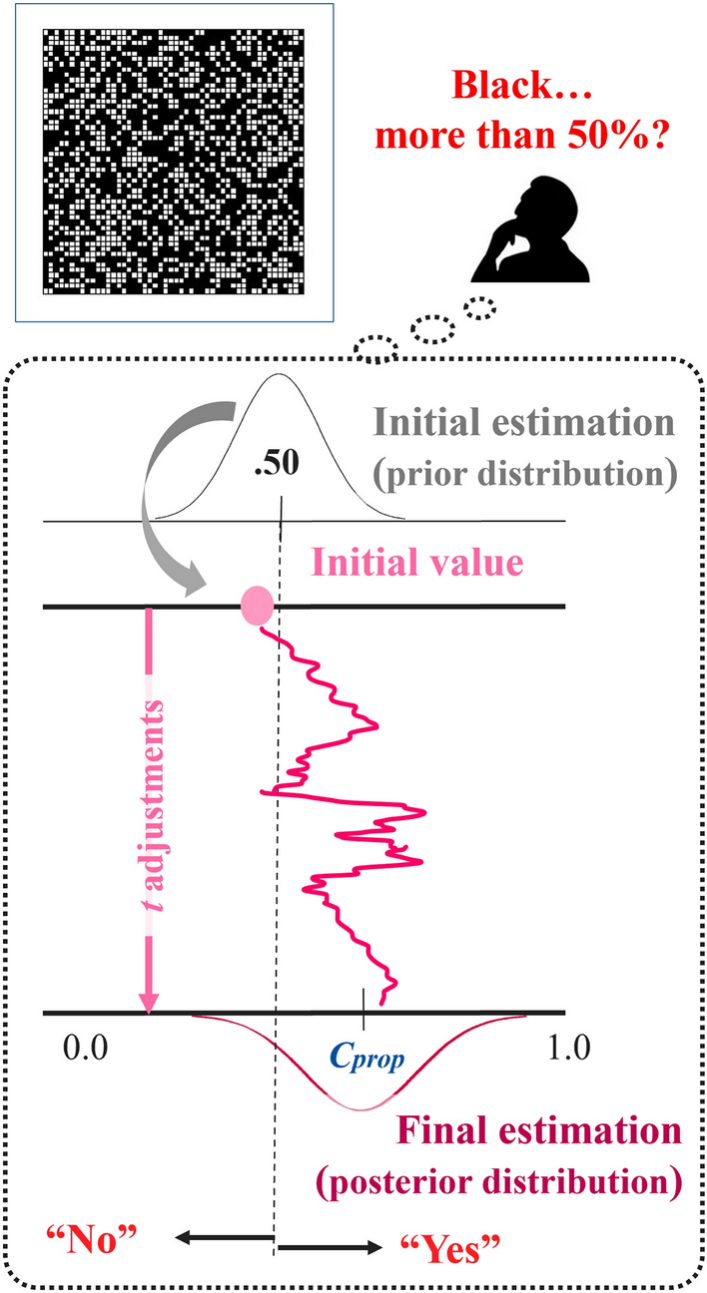
Schematics of computer simulations of the Grid Task. We assumed that a person was asked to judge whether the proportion of black grids was over 50%. The person’s first estimation was sampled from a normal distribution (mean = .50); and after *t*-time adjustments, a final estimation was provided. Our simulations iterated this procedure 1,000 times in one person, and assumed that 500 people conducted this task individually.

Consider the simple perceptual binary-choice task (called “grid task”; Fig. 1): A person is presented with a black-and-white grid stimulus and asked to judge whether the black grids compose more than 50% of the grids in the stimulus.

#### Procedure

We applied Lieder et al.’s framework of resource-rational analyses^17^ to the grid task. They explained the anchoring bias in terms of the resource rationality. Their model assumed that people’s adjustment of numerical estimation proceeds by repeatedly considering small changes to the current estimate. The proposed change is accepted or rejected probabilistically such that the change is more likely to be made the more probable the new value is and the less probable the current one is, following by Markov chain Monte Carlo (MCMC) method; more specifically, Metropolis–Hastings algorithm^28^. After sufficiently many adjustments, the estimate becomes correct on average. In the same time, however, each small adjustment is assumed to cost a certain amount of time. Then, the number of time steps of adjustments is chosen to minimize the expected value of the time cost of adjustment plus the error cost of the resulting estimate. According to Lieder et al., there are several advantages to consider such a computational model as a model for numerical estimation^17^. First, the success of MCMC methods in machine learning and statistics suggests that MCMC is well suited for describing many inference problems people face in daily life. Second, MCMC can explain important aspects of cognitive phenomena (e.g., category learning, the temporal dynamics of perception, and developmental changes in cognition). Finally, process models based on MCMC have the potential to explain why people’s estimates are variable^29^ and systematically biased^30^. It is assumed that participants repeatedly adjust their estimation over time also in our grid task. Therefore, we applied Lieder et al.’s computational modeling in this study.

In this grid task, we assumed that such adjustments were repeated 150 times: A person made estimation *v*_t_ at each time *t* = 0, 1, 2,…,150. The correct proportion, ${C}_{prop}$, of black grids in the presented stimulus was set as 0.55 (difficult) or 0.65 (easy), and the person’s estimation would be assumed to gradually approach ${C}_{prop}$ over time. Because a person is asked to judge whether the proportion of black grids is over 50% or not, the first value of estimation, *v*_1_, is assumed to be anchored at approximately 0.50. Specifically, it was sampled from a normal distribution (i.e., prior distribution) with a mean = 0.5 and *SD* = *b* (*b* represents the person’s strength of belief; fixed as 0.1). Hereafter, all normal distributions in our simulations were truncated for min = 0 and max = 1. We assumed that seeing the grid stimulus for a few seconds led to repeated adjustments of estimation. At each time *t*, a potential adjustment, $\delta$, was proposed by sampling from a normal distribution with a mean = 0 and *SD* = 0.05. Then, according to the Metropolis-Hasting framework, Either the previous value, *v*_t−1_, or adjusted value, *v*_t−1_ + $\delta$, was accepted as *v*_t_ by comparing probability densities *Prob*(*v*_t−1_) with *Prob*(*v*_t−1_ + $\delta$). Under a normal distribution with a mean = ${C}_{prop}$ and *SD* = *b*, the proposed value was always accepted if it was likely to make an estimation more probable (i.e., *Prob*(*v*_t−1_ + $\delta$) > *Prob*(*v*_t−1_)); even if the adjustment would be less probable (i.e., *Prob*(*v*_t−1_ + $\delta$) < *Prob*(*v*_t−1_)), it was also accepted with probability *Prob*(*v*_t−1_ + $\delta$)/*Prob*(*v*_t−1_); otherwise, the adjustment was rejected. Finally, it was regarded as correct if *v*_150_ > 0.50, and vice versa.

According to^17^, this algorithm generally ensures that regardless of which initial value the person starts from, the distribution of estimates *v*_t_ will eventually converge to around the true proportion if adjustments are repeated long enough (i.e., sufficiently increasing *t*). In other words, if sufficiently long adjustments were conducted, the person’s final judgment is likely to become accurate; while if adjustments stopped at insufficient time (e.g., due to time pressure), the person’s final judgment tends to be inaccurate.

Because human judgments are not expected to be constant (e.g., responses may vary even if the grid stimuli are the same proportion, because of grid pattern differences), the above procedure was iterated 1000 times per person. We defined the mean of the rate of correct judgments in these 1000 iterations for each time *t* as ${ThinkBenefit}_{t}$ (${ThinkBenefit}_{t}$ was re-scaled to arrange the maximum value as 1).

To account for the resource rationality, we added a thinking cost at time *t*, ${ThinkCost}_{t}$. Following^17^, we assumed that thinking costs increased linearly with time *t*. Regarding the traditional SAT, cognitive resources were not assumed and the maximum cost was 0.0 (i.e., ${ThinkCost}_{150}$= 0.0). In contrast, regarding resource rationality, cognitive resources were assumed and the maximum cost was over 0.0 (i.e., ${ThinkCost}_{150}$ > 0.0). In our resource rationality simulations, we set ${ThinkCost}_{150}$ to 0.50.

Finally, we defined the mean of the differences between the thinking benefits and thinking costs (i.e., ${ThinkBenefit}_{t}$ − ${ThinkCost}_{t}$) in 1,000 iterations for each time *t* as ${TotalBenefit}_{t}$.

Our simulations assumed that the above estimations in the grid task (i.e., 150-times adjustments * 1,000-times iterations) were conducted by 500 hypothetical individuals. For each of these individuals, we investigated when the peaks of the total benefits would be observed, in both cases not assuming thinking costs (SAT; i.e., ${ThinkCost}_{150}$ = 0.0) and cases assuming the thinking costs (resource rationality; i.e., ${ThinkCost}_{150}$ = 0.50).

### Results and discussion

**Fig. 2:**
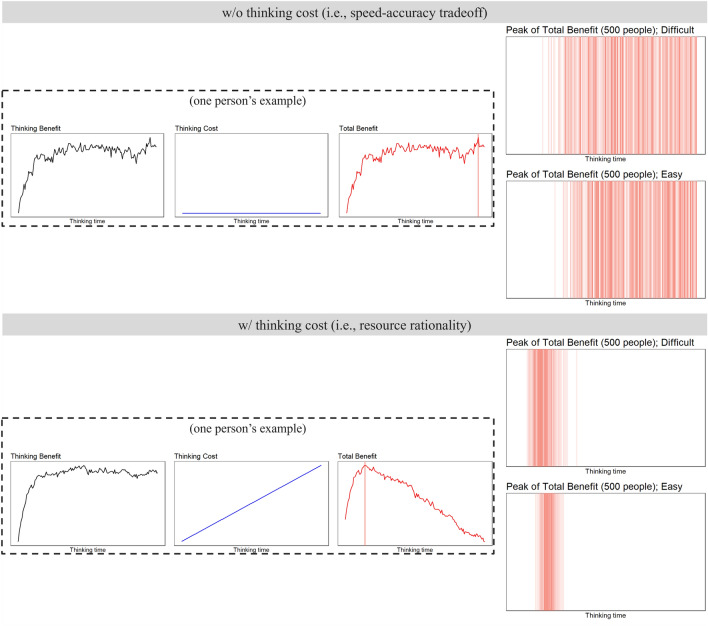
Results of computer simulations. The three panels inside the frames show examples of one person’s estimation. Red vertical lines denote the peak times of a total benefit. Upper: SAT simulations (i.e., a thinking cost was not assumed and a total benefit was equivalent to a thinking benefit). Lower: resource rationality simulations (i.e., a thinking cost continued to increase linearly and a total benefit decreased gradually after the peak). Panels outside frames show the peak times of total benefit observed across 500 individuals’ estimations. In both difficulty levels, the peaks of total benefit related to resource rationality were observed in narrower ranges and at earlier times than those in SAT.

First, we show an example of estimation for a single person (panels in the frames in Fig. 2). When the maximum thinking cost was 0.0, the total benefit was equivalent to the thinking benefit. This result can be explained by SAT; in fact, the curved lines depicted in the “Thinking Benefit” and “Total Benefit” panels in “w/o thinking cost” are a typical shape explaining SAT (i.e., the extent of the increase in benefit gradually declined as time went on, and the benefit did not decrease; e.g.^31,32^). However, when the thinking cost was introduced, the total benefit peaked at a certain point (vertical red line) and then gradually decreased. This result can be explained by resource rationality, which assumes limited cognitive resources. This assumption means that costs (e.g., cognitive workload) by thinking gradually increases over time. However, the improvement in accuracy plateaus at a certain point, so the total benefit (= thinking benefit – thinking cost) peaks at a certain point and then gradually decreases.

We also analyzed time points when the total benefit peaks were observed in the estimations by 500 hypothetical individuals (right-end panels of Fig. 2; intensity of the color denotes frequency). In SAT simulations, the peak times ranged widely, particularly from the middle to later time points. In contrast, in resource rationality simulations, the peak times were observed in narrow ranges, especially in earlier time points. Furthermore, such tendencies did not depend on task difficulty. Specifically, across both difficult and easy questions, the peak times of total benefit ranged widely in SAT (difficult: mean = 95.17, min = 23, max = 150; easy: mean = 100.2, min = 33, max = 150) and narrowly in resource rationality (difficult: mean = 23.16, min = 10, max = 51; easy: mean = 27.59, min = 17, max = 40).

Our results suggest the following two points. First, thinking longer is not always a good strategy. It is because the total benefit decreased in the later stages when thinking cost was considered. Even if the accuracy increases by thinking longer, it will be undesirable if people’s cognitive costs also increase. Second, there are likely to be peak times, regardless of difficulty. It is because we could obtain consistent results between difficult (black grid 55%) and easy (black grid 65%) questions. Of course, if the percentage of correct answers (i.e., proportion of black grids) differed more greatly between difficult and easy (e.g., 52% and 12%, respectively), peak times of total benefit might also have differed greatly between them (e.g. a peak would be observed much later in difficult questions while much earlier in easy questions). However, considering limited cognitive resources (i.e., an increase in thinking costs over time), it will be certain that a peak is observed in any case.

We should also point out that the SAT is inherently about costs, especially opportunity costs (i.e., time spent on one task is time not spent on another). Once such costs are taken into account, time costs may give rise to the kind of “total benefit” plots we observed in “w/ thinking cost” panel. In fact, the shape of function we showed was highly similar to the shape of function that Jarvstad et al. developed^33^, which was an efficiency functions for opportunity and environmental costs (e.g., how good it is to make a correct decision; how bad it is to make an incorrect decision). In standard sequential sampling models, such costs are not often plotted because they are not a key variable of interests (rather, key interests are often about which mechanisms can explain a particular response-time distribution; e.g.^34^). We directly focused on and theoretically showed the effects of costs (not “opportunity costs” but “thinking costs”). Our results can also be interpreted that an increase in opportunity costs through an increase in waiting time would lead to a shift in the optimal speed-accuracy trade-off and people seemed to be sensitive to.

One may argue that, although the current simulations assumed the prior belief as a unimodal distribution (mean = 0.5), a “bimodal” prior should be more appropriate because the correct answer was unclear whether black grids are more (or less) than 50% at the beginning of a question. Thus, we conducted additional simulations changing a prior belief assumption to a bimodal distribution, which had two peaks at 0.4 and 0.6 (i.e., we synthesized two normal distributions with mean = 0.4 and with mean = 0.6; *SD* = 0.05 in both distributions). Then, we observed the almost identical results in the current results. For details, see Supplementary information.

In summary, it is predicted that even if people think about a task for a long time, thinking benefits such as judgment accuracy will not continuously increase, and instead thinking costs (e.g., subjective irritation and conflicts) will become more likely to increase. It is also predicted that people’s judgment competence can be fostered with less thinking costs, by simply providing them an appropriately short waiting time before choosing alternatives (i.e., “wait short time” intervention that we propose). In the next section, we investigate these predictions through behavioral experiment.

## Study 2: Behavioral experiment

Next, we conducted behavioral experiment to empirically examine whether the resource-rational analyses based on computer simulations in Study 1 can well capture actual human behaviors.

### Methods

#### Participants

A total of 123 Japanese people participated in this study. Forty-two (*n*_men_ = 12, *n*_women_ = 30, *M*_age_ = 39.7, *SD*_age_ = 10.9), forty (*n*_men_ = 20, *n*_women_ = 20, *M*_age_ = 39.8, *SD*_age_ = 12.0), and forty-one (*n*_men_ = 20, *n*_women_ = 21, *M*_age_ = 35.0, *SD*_age_ = 10.5) participants were assigned to the 0 s, 1 s, and 2.5 s groups, respectively (note that in the 0 s group, three participants were excluded from the analyses because their data could not be appropriately recorded owing to technical errors). The total sample size was determined using G*Power^35^. We set the power to 0.9 and error probability to 0.05, and assumed effect size as 0.3–0.35 (medium level) in F-tests family, yielding a required sample size range of 108 (effect size 0.35) to 144 (effect size 0.3). Therefore, we recruited approximately 40 participants per group. All participants were registered with a Japanese company (Chuo Sato Inc.) as experimental monitors and provided written informed consent before participating in this study. No data that could identify participants was collected. The experimental protocols conformed to the Declaration of Helsinki and were approved by the Ethics Review Committee for Experimental Research at Otemon Gakuin University.

#### Tasks, procedure, and conditions

**Fig. 3:**
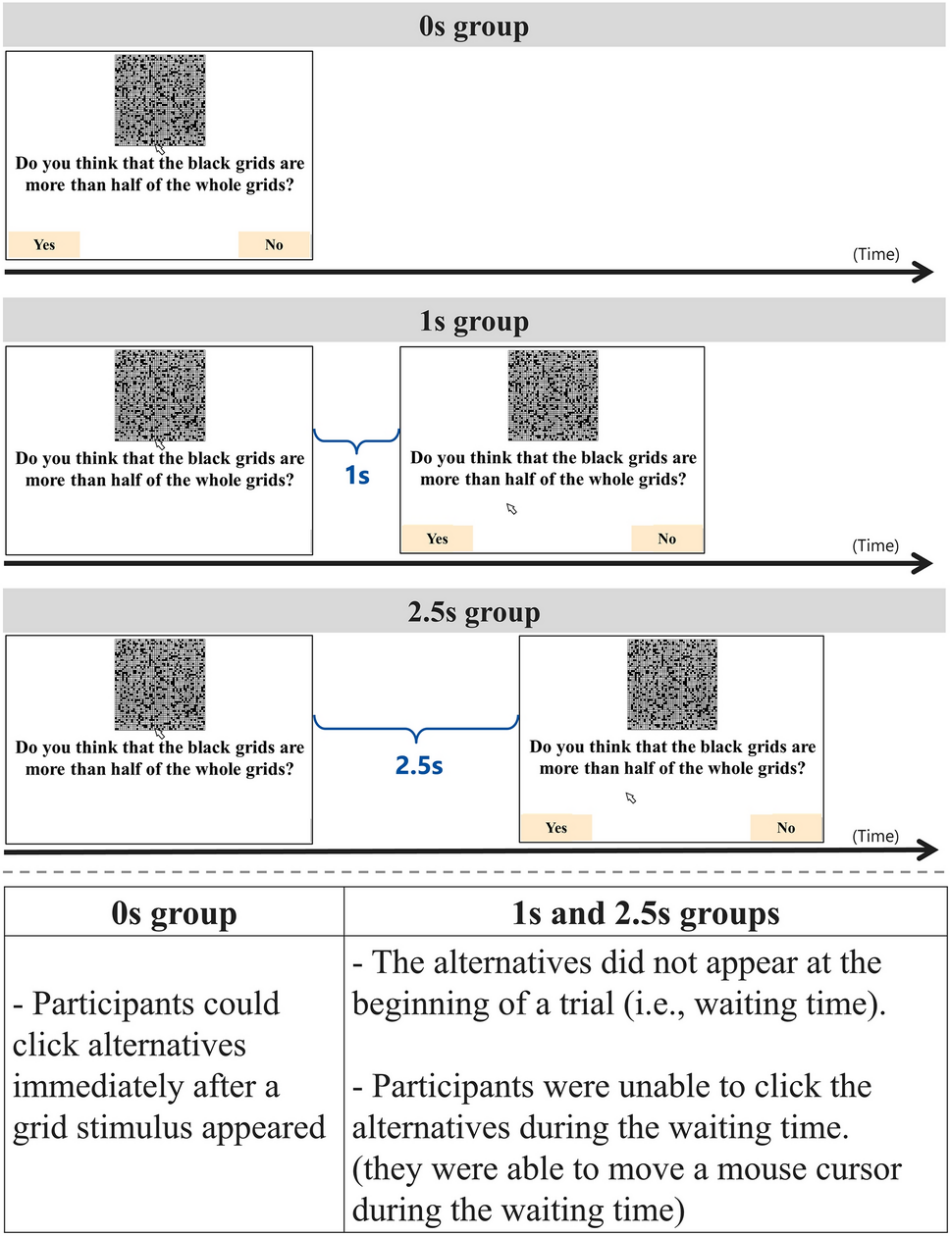
Schematics of behavioral experiment (general procedure of the grid task). Participants were asked to judge whether the black grids made up over 50% of the stimulus. Participants in the 0 s group could click one of the two buttons (“yes” or “no”) from the beginning of the trial. Participants in the 1 s and 2.5 s groups could not click the button during the first 1 s and 2.5 s of the trial, respectively. Note that even during the waiting time in the 1 s and 2.5 s groups, a stimulus constantly appeared on the screen and participants could move their mouse cursor.

The experiment were conducted using a computer and a mouse. We conducted the grid task described in Study 1 according to the following procedures. First, a fixation cross was presented for 0.5 s, which was followed by a black-and-white grid stimulus comprising 50 × 50 tiles. Participants were asked to judge whether the black grids composed more than 50% of the grids in the stimulus, and then to click the “yes” or “no” button presented at the bottom of the screen. After clicking a button, the “next” button was presented. Upon clicking the “next” button, a fixation cross appeared again, and this procedure was repeated (Fig. 3). One block included 40 questions, and nine blocks were conducted, meaning that each participant responded to 360 questions. We prepared four types of black-grids proportions for the stimuli (for grid examples, see Supplementary information): 45%, 55% (as difficult questions), 35% and 65% (as easy questions). For each participant, each stimulus appeared 90 times, and the order of grid stimuli presentation was randomized. Note that trials in which the response time was over 6 s were excluded from data analyses as outliers (2.1% of the total).

To investigate effects of waiting times, we experimentally manipulated the length of waiting time. Specifically, we set up the three groups: 0 s, 1 s, and 2.5 s groups (between-participants design). In the 0 s group, participants responded to the task normally. In the 1 s and 2.5 s groups, a waiting time was inserted at the beginning of the task (i.e., a time blank was placed before the two buttons appeared; see Fig. 3). During the waiting times, only yes and no buttons were hidden, and a stimulus was always presented on the screen and participants were free to move a mouse cursor. We expected participants provided with a waiting time (1 s or 2.5 s) to show higher judgment accuracy than those who were not (0 s), and those provided with an appropriately short waiting time (1 s) to experience lower thinking costs than those provided with an unnecessarily long waiting time (2.5 s).

#### Measurements of thinking costs (cognitive conflicts and subjective irritation)

We investigated participants’ thinking costs by assessing cognitive conflicts between the two alternatives and subjective irritation with the waiting times.

**Fig. 4:**
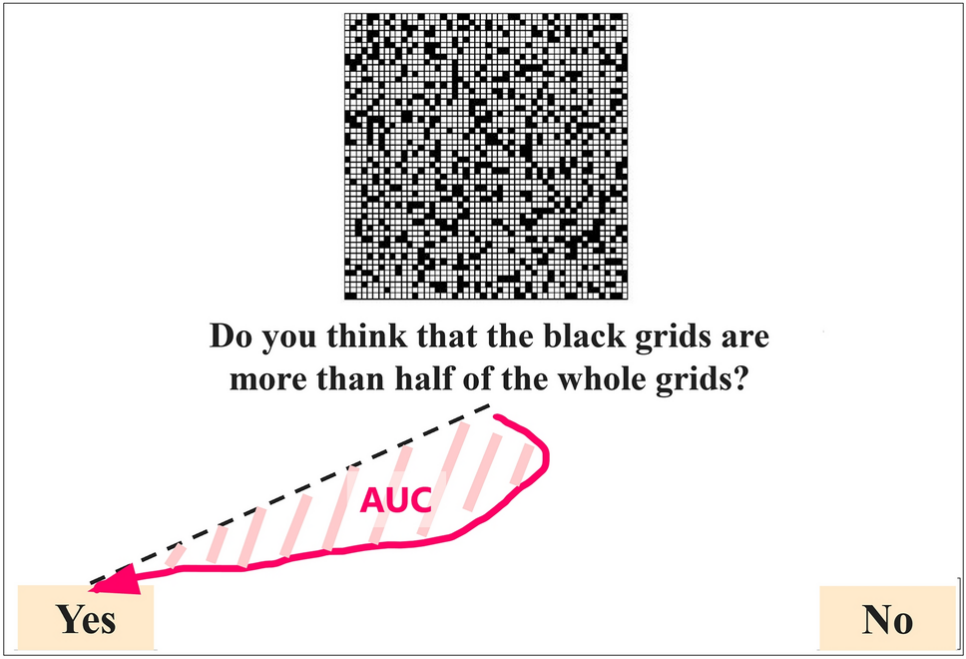
Example of the Area Under the Curve (AUC) using mouse tracking. It is an indicator of cognitive conflicts in deciding between alternatives and is defined as the area (colored area) between the actual trajectory (a curved pink arrow shows an example) and a straight trajectory linking trial onset and response termination (dotted line).

First, to investigate participants’ cognitive conflicts, we used mouse-tracking approaches. Previous studies indicated that the area under the curve (AUC) of mouse cursor movements can be used as an index of the extent of cognitive conflicts^36–38^. The AUC is defined as the amount of area between the actual trajectory and a straight trajectory from trial onset to response termination^39^ (Fig. 4). Trajectories that are more similar to a straight trajectory (i.e., smaller AUC) can be interpreted as reflecting fewer cognitive conflicts. In our experiment, we recorded the positions of the mouse cursor of each participant during each trial (i.e., from appearing a grid stimulus and to clicking a “yes” or “no” button). The initial position of the cursor in each trial was always at the center of the screen, and the x- and y-coordinates on the computer screen were recorded for each frame. That is, each time step had a corresponding x- and y- coordinate pair. Following previous studies^36,38,40,41^, we conducted time normalization of the recorded mouse trajectory because the response times differed between trials. The x- and y- coordinates of the mouse cursor in each trial were time-normalized to 101 time points using linear interpolation (i.e., 100 time-normalized equal distances). In other words, each trial was normalized to have the same number (101) of time steps. In calculating the AUC for each trial, we used R package “mousetrap”^40^.

Second, to investigate the effects of waiting times on subjective irritation, we conducted a questionnaire in the 1 s and 2.5 s groups. Specifically, after every block of the task, we asked participants to evaluate how irritated they got because of the time blank at the beginning of every trial, using a 101-point visual analog scale ranging from 0 (not at all) to 100 (very much). As there were nine blocks in the grid task, each participant rated their own irritation nine times. We regarded the maximum rating score across the nine blocks as the participant’s irritation. Note that we omitted participants who rated “0” in all blocks because we deemed them as inappropriate responses (as a result, *n*_1s_ = 37 and *n*_2.5 s_ = 37). We regarded a larger AUC (i.e., more cognitive conflicts) and larger rating scores (i.e., more subjective irritation) as larger thinking costs.

### Results and discussion

#### Thinking benefit

**Fig. 5:**
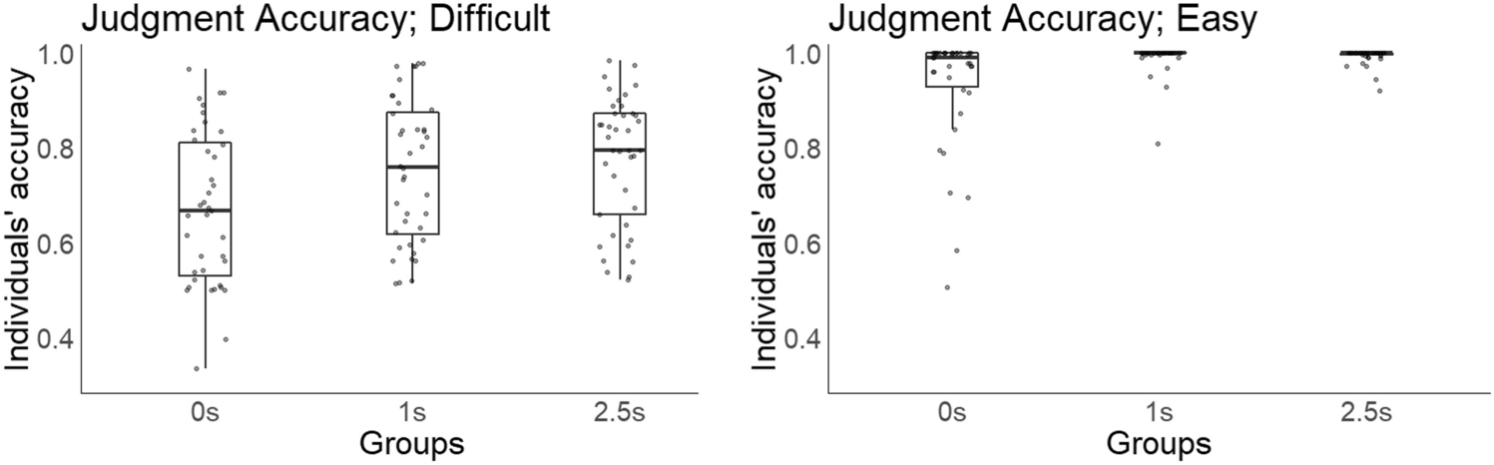
Judgment accuracy in the behavioral experiment. The term “individuals’ accuracy” here refers to the rates of correct judgments for each participant. The left and right panels show the results for difficult and easy questions, respectively. The x-axis shows the groups, and the dots denote individual data.

We regarded the rate of correct judgments of each participant (individuals’ accuracy) as thinking benefits. For each difficulty level, we calculated and compared individuals’ accuracy between the three groups (Fig. 5; for descriptive statistics, see the Supplementary information). In difficult questions, we found significant differences for accuracy, especially between the 0 s and 1 s groups and between the 0 s and 2.5 s groups (*M*_0s_ = 0.671, *M*_1s_ = 0.752, *M*_2.5 s_ = 0.775; *F*(2, 117) = 5.34, *p* = 0.006, ${\eta }^{2}$ = 0.08; 0 s vs. 1 s *p* = 0.034, 0 s vs. 2.5 s *p* = 0.007, 1 s vs. 2.5 s *p* = 0.492, Holm-adjusted pairwise comparisons). In easy questions, although ceiling effects were somewhat observed, we observed results similar to those for difficult questions (*M*_0s_ = 0.911, *M*_1s_ = 0.990, *M*_2.5 s_ = 0.993; *F*(2, 117) = 5.34, *p* < .001, ${\eta }^{2}$ = 0.13; 0 s vs. 1 s *p* < .001, 0 s vs. 2.5 s *p* < .001, 1 s vs. 2.5 s *p* = 0.890, Holm-adjusted pairwise comparisons). These results indicate that waiting times can lead to improve judgment accuracy (i.e., generate thinking benefits).

#### Thinking cost

**Fig. 6:**
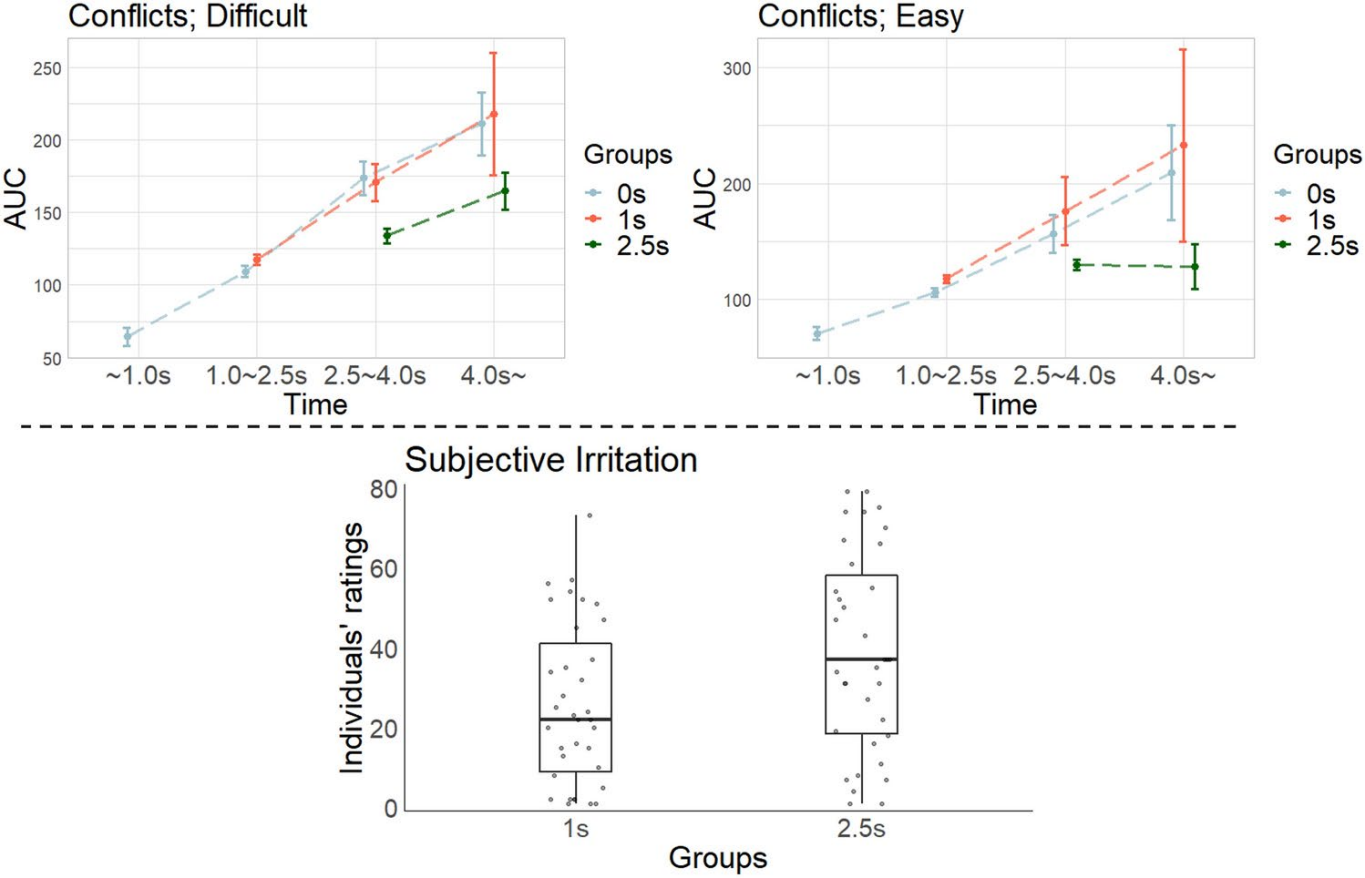
Cognitive conflicts and subjective irritation in behavioral experiment. Upper panels: AUC obtained from mouse tracking approaches. Blue, red, and green lines show the 0 s, 1 s, and 2.5 s groups, respectively. The left and right panels show the results for difficult and easy questions, respectively. The x-axis shows the response time categories (~ 1.0 s, 1.0–2.5 s, 2.5–4.0 s, and 4.0 s ~), and error bars denote 95% confidence intervals. Large dots connected by lines show the mean of AUC for each time category. Lower panel: ratings for subjective irritation regarding waiting times. The x-axis shows the groups, and the dots denote individual data.

Regarding cognitive conflicts, we first categorized response times into four categories (~ 1.0 s, 1.0–2.5 s, 2.5–4.0 s, and 4.0 s ~) and then calculated each trial’s AUC of mouse trajectories in these categories. We found that the AUC tended to increase linearly with time (Fig. 6, line plots), implying that participants’ cognitive conflicts tended to gradually and monotonically increase. This tendency corroborates our assumptions in Study 1 that thinking costs would linearly increase with time (see below the “Thinking Cost” panel in Fig. 2). In the easy questions of 2.5 s group, the mean of AUC did not increase with time. This might be due to the small number of observed responses and the large variance in responses. In easy questions, participants often made easy and quick judgments and most responses occurred immediately after the 2.5 s waiting time (number of observed responses: 6,828 in 2.5–4.0 s; 438 in 4.0 s ~), making the AUC variance larger in responses at 4.0 s ~ . As indicated by the 95% confidence intervals (error bars), large AUC were sometimes observed even in 4.0 s ~ (the upper bounds of 95% confidence interval: 134.3 in 2.5–4.0 s; 147.7 in 4.0 s ~). Note that, as shown in Fig. 6 upper two panels, the 2.5 s group showed lower AUC (less conflict) than either of the two other groups for comparable durations. It is possible that participants in the 2.5s group did not have to deeply engage in a task within the first 2.5 s and might experience less workload than those in the other groups. At the time 2.5 s had passed, the 0s and 1s groups had already had to engage the task for 2.5 and 1.5 s, respectively, which might generate more cognitive load in 0s and 1s groups than in 2.5s group.

Regarding subjective irritation, we compared individuals’ ratings between the 1 s and 2.5 s groups. We found that participants in the 1 s group experienced lower irritation with waiting time than those in the 2.5 s group (Fig. 6, boxplot; *M*_1s_ = 29.05, *M*_2.5 s_ = 41.78, *W* = 494.5, *p* = 0.039, Cliff’s delta = 0.277; Mann–Whitney *U*-test). These results suggest that providing an unnecessarily long thinking time is undesirable because it is likely to increase workers’ cognitive load. Because our focus at this time was the effects of waiting times, the 0 s group did not respond to a subjective irritation questionnaire. However, as some previous studies evaluated the effects of intervention (e.g., prompting people to think critically or about accuracy) between a “with-intervention” group and “without-intervention” group^42–46^, it will be more desirable to investigate subjective irritation also in the 0 s group. Therefore, we another (replication) study using the same questionnaire in all three groups. We then confirmed that subjective irritation in the 1 s group did not differ from that in the 0 s group and was lower than that in the 2.5 s group. For details, see the Supplementary information.

#### Total benefit

Finally, we regarded judgment accuracy with times as total benefits.

**Fig. 7:**
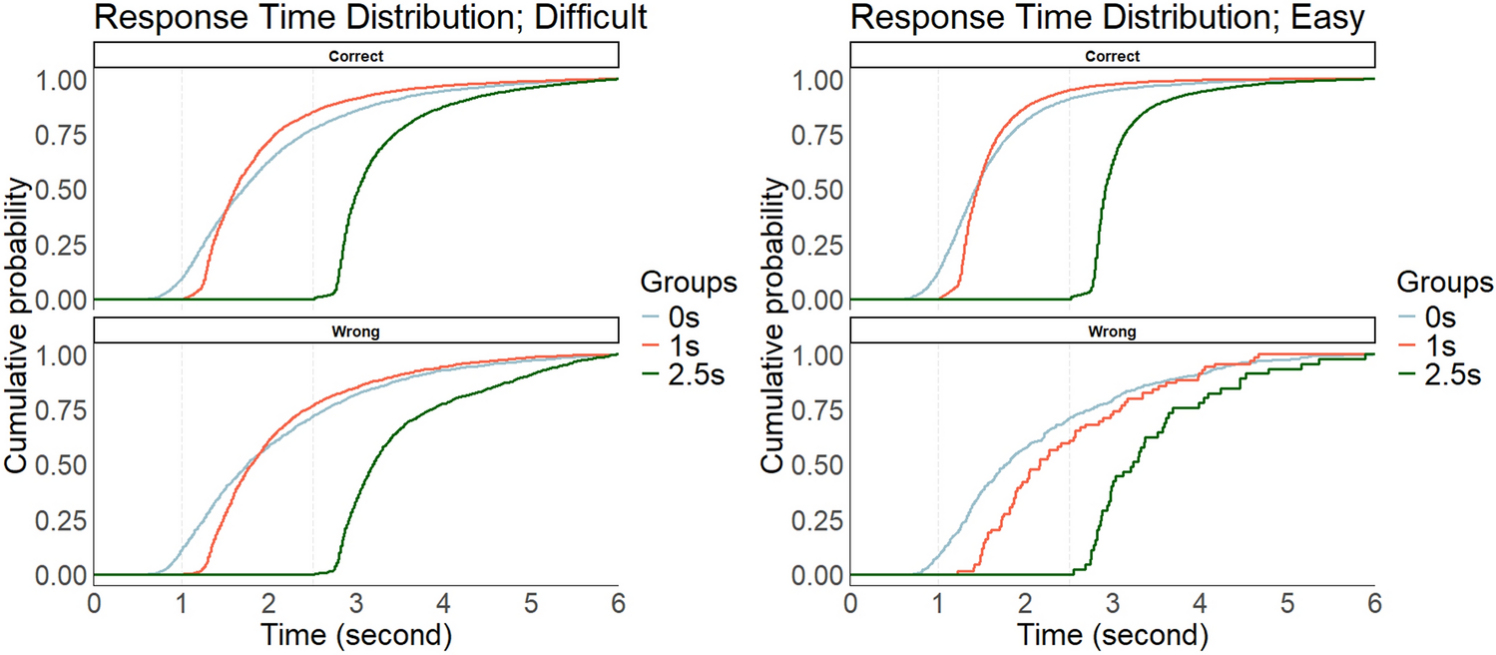
Response time distributions in the behavioral experiment. Blue, red, and green lines show the 0 s, 1 s, and 2.5 s groups, respectively. The graphs are depicted in the form of a cumulative distribution function. We separated the behavioral data into a 0.1-s bin. The x- and y-axes denote the response time (0–6 s) and cumulative probability, respectively. The left and right columns show the results in difficult and easy questions, respectively. The top and bottom rows show the results in correct and wrong trials, respectively.

Before describing results of total benefits, the distributions of response times were first shown in the form of a cumulative distribution function. In our analyses of response times, trials over 6 s were excluded as outliers, and the behavioral data were divided into bins of 0.1 s (i.e., the data from 0 to 6 s were divided into 60 bins). This analysis allows us to know whether effects of a waiting time on accuracy is due to an overall speed-up/slow-down of responses or due to a selective speed-up/slow-down at specific points in the cumulative distribution function^47^. In our analyses, we divided the trials into difficult (i.e., black grid 45% or 55%) and easy (i.e., black grid 35% or 65%) trials, and calculated the cumulative distribution of response times for correct and false responses in each group (Fig. 7). For the descriptive statistics of response times, see Supplementary information. The results suggest that many correct judgments tended to be observed in the early stages: Specifically, at approximately 1 s in the 0 s group, and immediately after the waiting times in the 1 s and 2.5 s groups. Such tendencies were common for both difficult and easy questions. In addition, false responses (compared to correct responses) tended to be distributed in the relatively later stages (i.e., right side of the graph), which implies that taking a long time does not necessarily lead to accurate judgments.

**Fig. 8:**
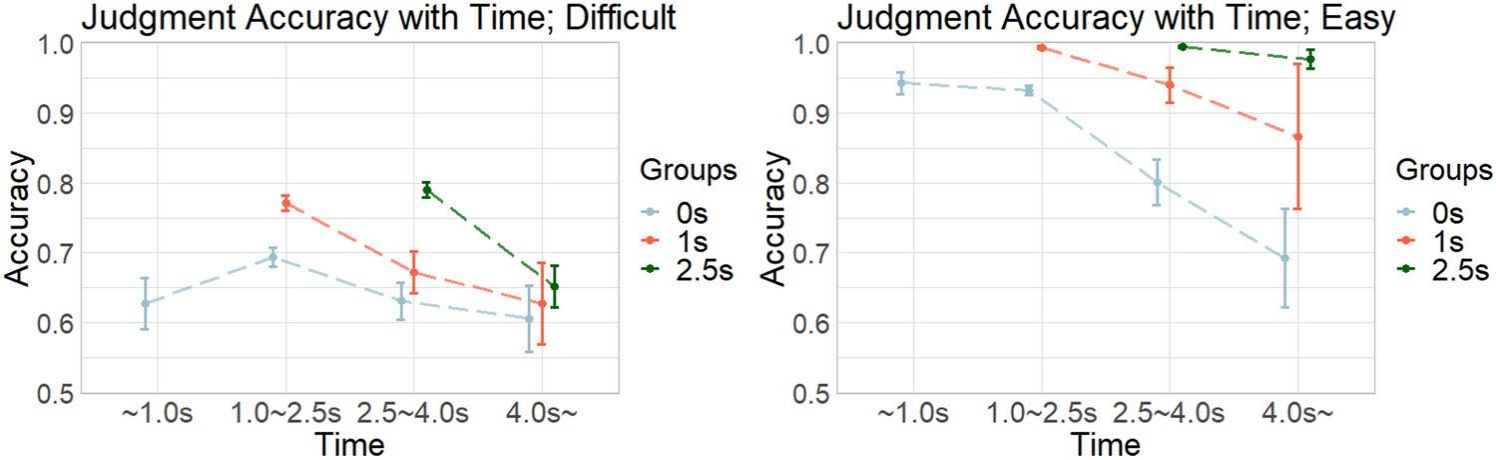
The changes of judgment accuracy with time in the behavioral experiment. Blue, red, and green lines show the 0 s, 1 s, and 2.5 s groups, respectively. The left and right panels show the results for difficult and easy questions, respectively. The x-axis shows the response time categories (~ 1.0 s, 1.0–2.5 s, 2.5–4.0 s, and 4.0 s ~), and error bars denote 95% confidence intervals. Dots connected by lines show the mean of judgment accuracy for each time category.

Then, we analyzed the rate of judgment accuracy within the four response time categories (Fig. 8), and found that judgment accuracy generally decreased as time elapsed and did not always improve at the late stages (e.g., later than 2.5 s) in all groups. Importantly, for the difficult questions in the 0 s group, judgment accuracy peaked at 1.0–2.5 s, suggesting that there is an appropriate length of thinking time (not too long but not too short; 1 s in this case) that enables to make judgments more accurately. In fact, the accuracy of the 1 s group was higher than that of the 0 s group (see Fig. 5), which corroborates this finding.

One may argue that these results can be explained in terms of stimulus encoding in sequential sampling models^15^. That is, additional thinking time can generally improve the quality of stimulus encoding, and thus the increase of accuracy in early stages (e.g., 1–2.5 s) was observed by such better stimulus encoding. Similarly, the greater accuracy in 1 s group can be interpreted due to, for example, better attentional engagement, lower noise, and so on. However, we would like to emphasize that the important point is that an “optimal” thinking time (a peak of accuracy) was observed also in actual behaviors. As described earlier, we consider that our model based on resource rationality framework will not always be in apposition to SAT framework, and thus we do not perfectly deny the SAT framework. Although our model development and behavioral evidence can validate our predictions about the effects of “wait short time” intervention, we admit that these results also offer alternative explanations and complementary (i.e., the effects of waiting times on accuracy, irritation, AUC etc. might be multifactorial). Regardless of the framework, however, we believe that waiting a short time is demonstrated to be an effective intervention based on the behavioral experiment.

It can also be considered that participants took longer time simply because the trial was difficult. We could not completely exclude this possibility within the current behavioral data. However, even when trials were separated by two difficulty levels (“difficult” as 45 and 55% stimuli, and “easy” as 35 and 65% stimuli), there consistently observed similar tendencies that accuracy decreased over time, as shown in Fig. 8. Thus, our behavioral results were unlikely to be observed due to the levels of difficulty.

Study 2 has thus empirically confirmed the resource-rational behaviors that were theoretically demonstrated by computer simulations in Study 1. Specifically, affording workers an appropriately short waiting time could work as a resource-rational intervention to maximize workers’ thinking benefits with lower thinking costs.

## General discussion

As suggested by SAT, longer thinking is believed to generate better benefits for workers engaging in judgment tasks, such as increase accuracy. However, as suggested by resource rationality, human cognitive resources are limited and thus a longer thinking will also increase thinking costs, such as cognitive load. This suggests that longer thinking will not always generate better benefits (i.e., the benefit will peak at a certain time point and then decrease). Based on the resource rationality framework, we proposed a simple intervention to improve workers’ judgment accuracy: Introducing an appropriately short waiting time prior to presenting alternatives. In Study 1, we used computer simulations to theoretically demonstrate that when assuming limited cognitive resources, the total benefit (i.e., the trade-off between thinking benefits and thinking costs) peaks at an early stage regardless of task difficulty. Then, Study 2 empirically confirmed these theoretical findings through behavioral experiment. Specifically, waiting times (1 s or 2.5 s) improved judgment accuracy, but an unnecessarily long time (2.5 s) was likely to increase subjective irritation. These findings show that providing workers with an appropriately short time (1 s in this case) could be an effective intervention for improving accuracy with a lower cognitive load.

One of the main advantages of our proposed boost is its high level of simplicity: Researchers only need to afford workers an appropriate thinking time without any explicit prompts. Furthermore, waiting a short time can strike a balance between allowing sufficient cognitive processing and minimizing cognitive load. It is considered that an appropriately short time wait (about 1 s in this study) will provide an “optimal” arousal level for generating better performance, known as a foreperiod effect^48,49^. The foreperiod effect refers to how response time and error rate are modulated by a duration between a warning cue and a stimulus (this duration is called a foreperiod). Participants can utilize the relationship between them to prepare for the imperative stimulus before it appears. In a simple choice task, plotting response time as a function of foreperiod typically yields a U-shaped curve: As foreperiod increases, response time first decreases, reaching its lowest point on the curve (at about 250-ms foreperiod) and then increases as the foreperiod gets longer^50^. The results in our study are consistent with this effect. We believe that we could obtain theoretical and empirical evidence of the effectiveness of “wait short time” intervention in this study. With online workers, one possible problem is that the time blanks are often predetermined in click experiments and therefore cannot be varied voluntarily. As a result, this intervention may not be implementable as a psychohygienic measure in online worker situations. For more practical implications, it may be worth considering and investigating the implementation of micro-breaks in the work routine, such as “regular vs. voluntary” breaks^51^.

### Limitations and future studies

Our intervention is highly simple and cost-effective, but it remains several open questions whether and to what extent the effect of our proposed intervention is universally applicable.

First, this study conducted only one perceptual judgment task (grid task); hence it remains unclear the generalizability of the intervention. Future studies should investigate whether the proposed intervention is also effective for other choice tasks that may be conducted by actual crowdsourcing workers (e.g., whether there is an abnormal symptom in a medical image).

Second, an “optimal” waiting time (about 1 s in this study) will vary depending on task types. For example, in a complex multiact task such as chained arithmetic (the processing time will be significantly longer; e.g., 1 + 1 = 2, 1 + 1 + 1 = 3, 1 + 1 + 1 + 1 = 4, etc.), the waiting interval may be absorbed into the overall processing time of the task. We again emphasize that what is important is not the specific length of thinking time (e.g., 1 s), but the existence of an “optimal”, appropriately short time for simple judgment tasks and providing a longer time will become an ineffective intervention. We believe that there will be an optimal thinking time for generating better performance with minimum cognitive load, even in complex tasks described above (the optimal time may be much longer than 1 s).

Third, related to the second point, the resource-rational model and stimulus-encoding model should be more rigorously compared. This study did not identify a model that could better explain people’s behaviors because our focus was to propose a simple, low-cost, and resource-rational intervention to improve judgment accuracy. To investigate the more detailed cognitive processes such as whether participants really used the resource-rational or stimulus-encoding strategy, future study will need to collect additional data such as arousal and preparation (e.g., varying time blanks across several experiments systematically^48^). If the observed data is related to resource rationality, we should discuss the reward structure and decision environment. The amounts of rewards and costs will affect what is rational to do (e.g., if making an inaccurate decision is very costly [e.g., may lead to death], decision-time should increase to emphasize choice accuracy over speed^52^. We believe that people have some flexibility in assigning resources, as some decision-making studies have suggested^53,54^. However, we did not introduce such parameters in the current simulation and did not provide incentives or any incentives in the behavioral experiment. If such parameters are considered, we may be able to provide further explanations of differences between SAT and resource rationality.

Fourth, there is room for consideration of differences in the subjective cognitive load between pre-test and post-test with more widely used measures. Regarding cognitive load, we analyzed the behavioral indicators (AUC) as well as asked participants to rate subjective workload. However, the subjective evaluation was only in post-test (i.e., after every block of the task) and our original question was used (i.e., irritation for a time blank). It will be needed to consider collecting pre-test to post-test measures such as engagement, distress, and worry as fundamental dimensions of subjective state in performance settings ^55^.

In conclusion, this study emphasizes the importance of considering the trade-off between thinking benefits and costs in the contexts of SAT and designing resource-rational interventions to optimize workers’ benefits.

## Supplementary Information

Supplementary Information.

## Data Availability

The data, R code, and Supplementary information are available at https://osf.io/w6hsv/?view_only=ce4479187111484fbd73f7a2673084b5.
