# Supplementary material for: Pause before action: Waiting short time as a simple and resource-rational boost
Source: Sci Rep. 2025 Feb 5;15:4362. doi: 10.1038/s41598-025-87119-z (PMC11799143; doi:10.1038/s41598-025-87119-z)
Supplement: Supplementary file 1 — Supplementary Information. [file 41598_2025_87119_MOESM1_ESM.pdf]

Appendix

Pause before action: Waiting short time as a simple and resource-rational boost

Example of the resource-rational approach: Numerical estimations (Lieder et al., 2018)

Using computational modeling and simulations, [1] showed that the anchoring bias in numerical estimations (i.e., prior numerical information affecting subsequent numerical estimations) reflects people's rational use of cognitive resources. The assumptions are briefly summarized as follows: In a numerical estimation task, individuals make the first estimation based on a presented anchor (i.e., prior numerical information). They then repeatedly adjust their estimations and finally make a final estimation. By repeating estimations, the posterior distribution of the final estimation can be acquired.

Lieder et al.'s model assumed that the number of optimal steps, t^*, was selected to minimize the expected value of the sum of the error cost of the final estimate and the time cost (or thinking cost) of the adjustments:

$$t^* = arg \min_t \left[E_{Q(\widehat{x_t})} \{cost(x, \hat{x}) + \gamma * t\} \right]$$

where $\hat{x}$ and $Q(\widehat{x_t})$ were the final estimation and posterior distribution of estimations after t-time adjustments, respectively; x was the (unknown) true value; $cost(x, \hat{x})$ was the error cost (i.e., absolute deviation between x and $\hat{x}$); and γ was a thinking cost per adjustment. In their computer simulations, the adjustments were assumed to be repeated for a certain time based on Markov chain Monte Carlo (MCMC) methods; specifically, the Metropolis-Hastings algorithm [2]. Importantly, [1] introduced a time cost (thinking cost) based on the resource rationality framework. Specifically, an increasing number of adjustments (i.e., thinking for a longer time) may generally lead to a reduction in errors (i.e., increase in judgment accuracy), but this should also linearly increase the thinking costs. Thus, the total benefit would decrease after t^*-time adjustments. [1] showed that the optimal number of adjustments, t^*, was much smaller than the number of adjustments required to eliminate the anchoring biases. They argued, thus, that there should be a point that reflects an "optimal resource allocation" under the trade-off between judgment accuracy and time cost.

Example of grid stimuli

For our grid task, 40 grid patterns were prepared for each of the four percentages of black grids, totalizing 160 stimuli. The order in which the stimuli were presented in the experiments was randomized, and the proportion of presented stimuli was counterbalanced between participants. We regarded stimuli with 45% and 55% of the grids as black grids as difficult questions, and those with 35% and 65% of the grids as black grids as easy questions. The experiments were performed using PsychoPy 3.0.

Examples of grid stimuli used in the behavioral experiments.

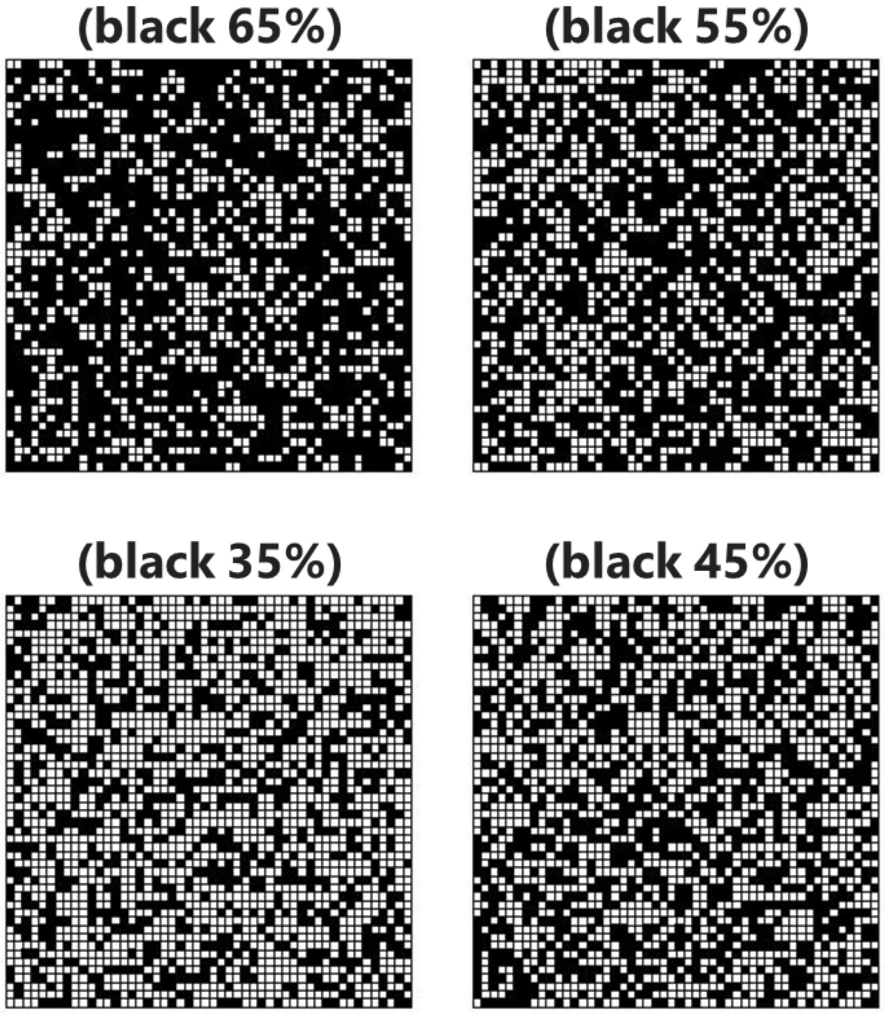

Note. A total of 40 grid patterns were presented for each percentage of black grids. The stimuli with Black 65% and 35% of the black grids (left column) were regarded as easy, and those with 55% and 45% of the black grids (right column) were as difficult.

Results of additional simulations: “Bimodal” prior

In the simulation study, we assumed the prior belief as a unimodal distribution (mean = 0.5). However, a “bimodal” prior should be more appropriate because the correct answer was unclear whether black grids are more (or less) than 50% at the beginning of a question. Thus, we conducted additional simulations changing an assumption of the prior belief to a bimodal distribution. We synthesized two normal distributions, with mean = 0.4 and with mean = 0.6. Both *SD*s were 0.05, which was half the value of the original distribution (a normal distribution with a mean = 0.5 and *SD* = 0.1; see the main text). Then, we observed the almost identical results in the current results.

w/o thinking cost (speed-accuracy trade-off)

one person’s example

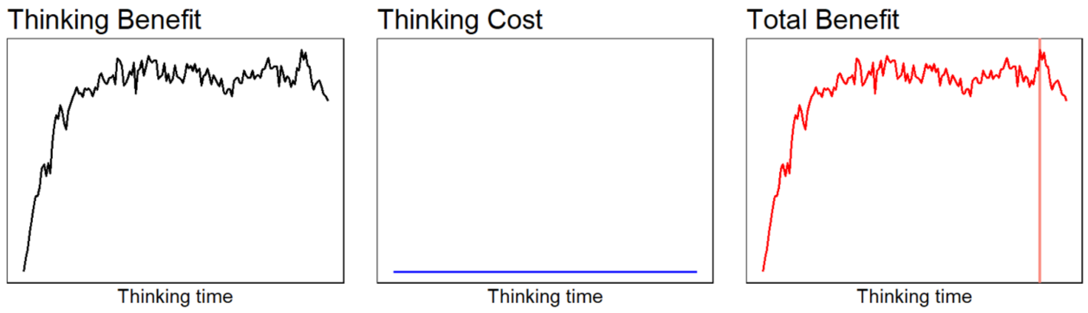

peak times of total benefit in 500 people; difficult

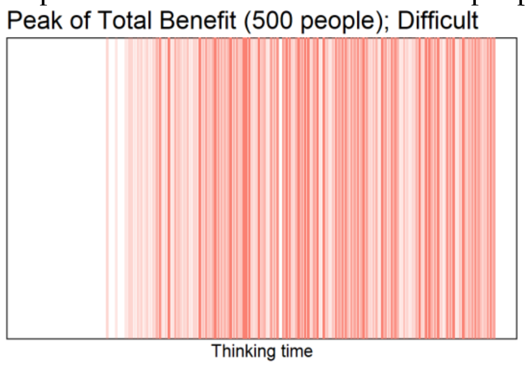

peak times of total benefit in 500 people; easy

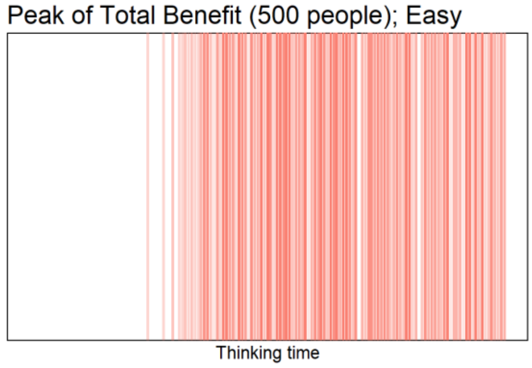

w thinking cost (resource rationality)

one person’s example

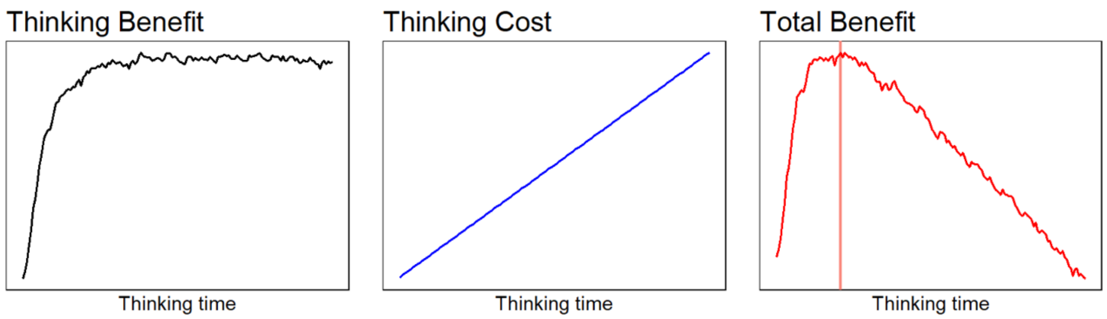

peak times of total benefit in 500 people; difficult

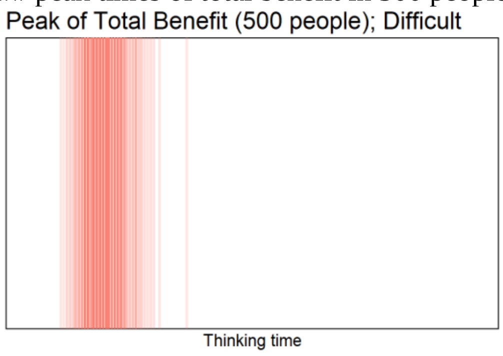

peak times of total benefit in 500 people; easy

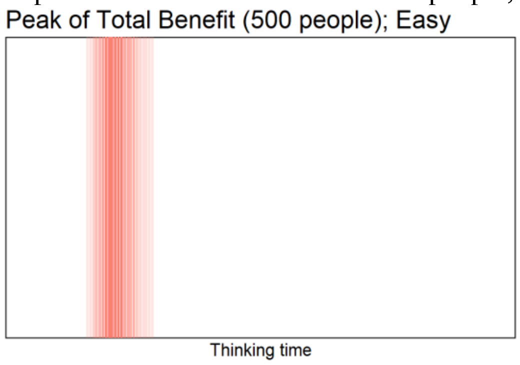

Descriptive statistics of the behavioral experiments: Judgment accuracy

Distributions of individuals' judgment accuracy (i.e., the rates of correct judgments for each participant) by question difficulty.

Distribution for all questions

	Min.	1st Qu.	Median	Mean	3rd Qu.	Max.
0s group (N = 39)	.253	.731	.837	.792	.900	.980
1s group (N = 40)	.643	.805	.876	.869	.931	.991
2.5s group (N = 41)	.710	.829	.900	.880	.934	.993

Distribution for difficult questions

	Min.	1st Qu.	Median	Mean	3rd Qu.	Max.
0s group (N = 39)	.336	.530	.667	.671	.811	.966
1s group (N = 40)	.514	.618	.759	.752	.874	.978
2.5s group (N = 41)	.522	.659	.794	.775	.872	.983

Distribution for easy questions

	Min.	1st Qu.	Median	Mean	3rd Qu.	Max.
0s group (N = 39)	.193	.919	.989	.911	1.00	1.00
1s group (N = 40)	.809	1.00	1.00	.990	1.00	1.00
2.5s group (N = 41)	.919	.994	1.00	.993	1.00	1.00

Descriptive statistics of the behavioral experiments: Response time

Distributions of response times. The units are seconds. We excluded trials in which the response time was longer than 6s as outliers.

Distribution for all questions

	Min.	1st Qu.	Median	Mean	3rd Qu.	Max.
0s group (N = 39)	0.547	1.20	1.53	1.81	2.13	5.94
1s group (N = 40)	1.02	1.33	1.52	1.75	1.90	5.99
2.5s group (N = 41)	2.52	2.84	2.97	3.20	3.29	5.99

Distribution for difficult questions

	Min.	1st Qu.	Median	Mean	3rd Qu.	Max.
0s group (N = 39)	0.598	1.25	1.72	2.02	2.48	5.98
1s group (N = 40)	1.02	1.38	1.65	1.94	2.15	5.99
2.5s group (N = 41)	2.52	2.87	3.05	3.34	3.52	5.99

Distribution for easy questions

	Min.	1st Qu.	Median	Mean	3rd Qu.	Max.
0s group (N = 39)	0.547	1.16	1.45	1.65	1.88	5.97
1s group (N = 40)	1.02	1.32	1.45	1.61	1.70	5.89
2.5s group (N = 41)	2.52	2.83	2.92	3.09	3.14	5.99

Subjective irritations including 0s group (additional, replication study)

In addition to the present study, we conducted another online study using the same procedures as reported in Study 2, with some minor differences (as in Study 2, the experimental protocols conformed to the Declaration of Helsinki and were approved by the Ethics Review Committee for Experimental Research at Otemon Gakuin University). Although the subjective irritation questionnaire was used only in the 1s and 2.5s groups in Study 2 (to focus on effects of waiting time on thinking costs), this replication study applied the questionnaire to all three groups. As in Study 2, participants in this additional study were asked to evaluate their subjective irritation with the waiting time at the beginning of every trial using a 101-point visual analog scale ranging from 0 (not at all) to 100 (very much).

The additional study allowed for comparisons of subjective irritation between groups with and without waiting time. Results showed that subjective irritation in 1s group (n = 62) did not differ from that in 0s group (n = 55), and was lower than that in the 2.5s group (n = 60) (M_{0s} = 20.29, M_{1s} = 26.58, $M_{2.5s}$ = 46.07, $F(2, 174) = 16.73$, $p < .001$, $\eta^2 = 0.16$; Holm-adjusted pairwise comparisons, 0s vs. 1s $p = .18$, 0s vs. 2.5s $p < .001$, 1s vs. 2.5s $p < .001$). This result indicates that an appropriately short waiting time (1s) is unlikely to increase the workers' thinking costs, whereas an unnecessarily long waiting time (2.5s) is likely to increase these costs.

Subjective irritation in our additional study.

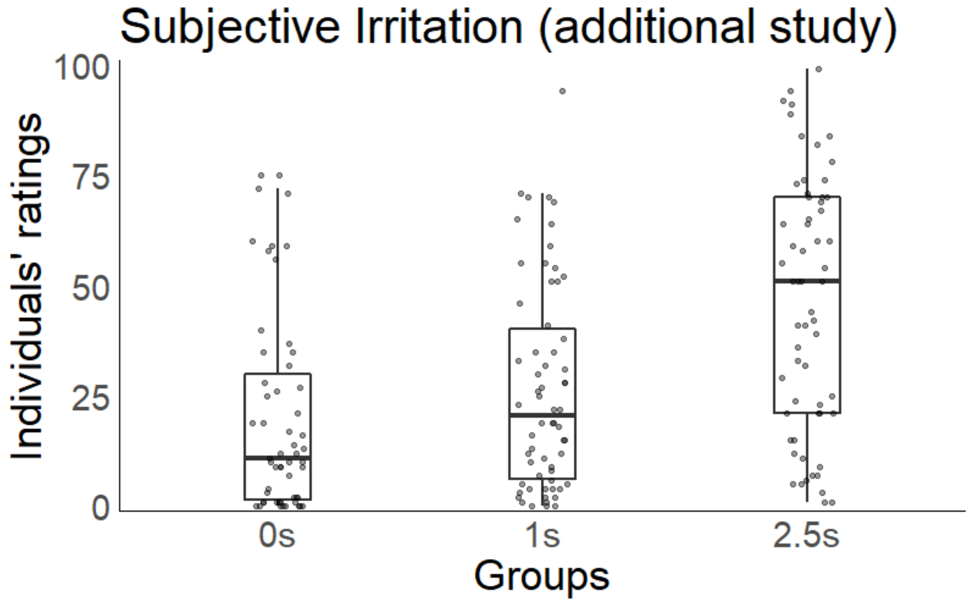

Note. Dots denote individuals' rating scores.

References

1. Lieder, F., Griffiths, T. L., Huys, Q. J. M. & Goodman, N. D. The anchoring bias reflects rational use of cognitive resources. *Psychon Bull Rev* 25, 322–349 (2018).
2. Hastings, W. K. Monte carlo sampling methods using Markov chains and their applications. *Biometrika* 57, 97–109 (1970).
